# Supplementary material for: Lived experience of young people with epilepsy in Bahir Dar city government hospitals, Ethiopia, a qualitative interview study
Source: PLoS One. 2023 Jan 23;18(1):e0278936. doi: 10.1371/journal.pone.0278936 (PMC9870136; doi:10.1371/journal.pone.0278936)
Supplement: Attachment — Submitted filename: Review manuscript Number PONE-D-22-19800.docx [file pone.0278936.s003.docx]

**Manuscript Number PONE-D-22-19800R1**

**Lived Experience of Young People with Epilepsy in Bahir Dar City Government Hospitals, Ethiopia, 2021; Phenomenological study**

Comments and recommendation.

Thank you so much for giving me an opportunity to review this interesting paper. Below are my comments:

**Design:**

***Population and sample:***

1. Please explain how were study participants selected? It was purposive, convenience, etc.
2. I suggest to provide information regarding inclusion and exclusion criteria.

***Data collection***

3. In which interview data saturation was achieved?
4. Were transcripts returned to participants for comment or clarification?

***Data analysis***

5. Since it is a phenomenological study, please make clear the data analysis method. If thematic analysis, which thematic data analysis steps was adopted?

***Results***

6. In Theme 2 (Subtheme 2) - Troublesome somatic symptoms should be put in theme 1: Psycho-social experience
