# Supplementary material for: Lived experience of young people with epilepsy in Bahir Dar city government hospitals, Ethiopia, a qualitative interview study
Source: PLoS One. 2023 Jan 23;18(1):e0278936. doi: 10.1371/journal.pone.0278936 (PMC9870136; doi:10.1371/journal.pone.0278936)
Supplement: Attachment — Submitted filename: Response to Reviewers.docx [file pone.0278936.s004.docx]

**RESPONSE TO EDITOR AND REVIEWERS**

**RESPONSE TO EDITOR**

**RESPONSE: Thank you for coordinating the review process and fruitful comments. We have revised the manuscript and addressed Reviewer’s comments.**

**Journal Requirements:**

**COMMENT:** Please review your reference list to ensure that it is complete and correct. If you have cited papers that have been retracted, please include the rationale for doing so in the manuscript text, or remove these references and replace them with relevant current references. Any changes to the reference list should be mentioned in the rebuttal letter that accompanies your revised manuscript. If you need to cite a retracted article, indicate the article’s retracted status in the References list and also include a citation and full reference for the retraction notice.

**RESPONSE: Thank you for your fruitful comment, we have reviewed our reference list and we can make sure for you that it our reference list is complete and correct**

**RESPONSE TO REVIEWER 1:**

**Comments to the Author**

Reviewer #1: Thank you for all efforts in amending the manuscript. Most of the reviewer comments have been addressed. But there are still some issues to ameliorate. The result and the study performance are not a phenomenological study.
Some suggestions, in order to amend the manuscript.

**RESPONSE: We thank the reviewer for kind words. We have revised our manuscript based on the comments as described below.**
 **REVIEWER COMMENT:** The title; Lived Experience of Young People with Epilepsy in Bahir Dar City Government Hospitals, Ethiopia, 2021; Phenomenological study-
change it to Lived Experience of Young People with Epilepsy in Bahir Dar City Government Hospitals, Ethiopia, A qualitative interview study

**RESPONSE: Thank you for your fruitful comment, we have revised our title by changing it by to Lived Experience of Young People with Epilepsy in Bahir Dar City Government Hospitals, Ethiopia, A qualitative interview study**

**REVIEWER COMMENT:** This study is about the lived experiences, so it is not necessary to point out 2021. (The period could be presented in data collection)

**RESPONSE: Thank you for your fruitful comment, we have revised it by removing 2021 from the title**

**REVIEWER COMMENT:** I am s ad to say, but this could never ever be a phenomenological study an interview guide with 14 demographic questions and then 17 epilepsy question- far too many to be a phenomenological study. The interviews lasted between 30-60 minutes, with all these questions there is not an in- deep interview and the questions are directing the interviewee. But a qualitative interview study with a Thematic Analysis could be OK. There are no in-deep interviews, there is semi-structured interviews. Revise it throughout the whole manuscript.

**RESPONSE: Thank you for your fruitful comment, we have revised it as a qualitative interview study with a Thematic Analysis**
**REVIEWER COMMENT:** Delete this sentence; Participants gave their verbal informed consent to share their data purely for scientific purposes without disclosing their true name, from the Rigor and trustworthiness—you have it un the Ethical section.

**RESPONSE: Thank you for your fruitful comment, we have revised it by deleting that paragraph from the Rigor and trustworthiness.**
**REVIEWER COMMENT:** In the Discussion, you start with This study discussed the psychosocial distress of young people with epilepsy in three interrelated categories. These include feeling about living with epilepsy,
social isolation, and academic challenges. The result is presenting 5 themes???? So, what is this? There is a need of correcting some text here in the discussion

**RESPONSE: Thank you for your fruitful comment, we have revised it as**

**This study discussed the lived experience of young people with epilepsy in five interrelated themes. These include psycho-social experience, physical experience, economic experience, health care related experience and Coping strategies.**
**REVIEWER COMMENT:** Limitation, please don’t talk about bias in a qualitative study. What the interviewee is telling us, is what they have experienced, it is their reality. Yes, qualitative findings cannot be generalized but they can be transferable, Guba and Lincolns transferability, if we as readers can judge the audit trail and understand the process.
Methodological issues are about the researchers” bias,” maintaining neutral and being aware of own preconceived ideas. Please re-write and correct this

**RESPONSE: Thank you for your fruitful comment, we have revised it based on your comment**.
**REVIEWER COMMENT:** The references are updated, but still there is no reference regarding phenomenology, so why not stay with the references—a qualitative interview study, analyzed with thematic analysis approach.

**RESPONSE: Thank you for your fruitful comment, we have revised it as a qualitative interview study and stayed with the references which support this paragraph**
**REVIEWER COMMENT:** I hope these comments will encourage you to make the amendments and thereby have a manuscript suitable for publication
I wish you the best

**RESPONSE: Thank you for your Kind Words**

**RESPONSE TO REVIEWER 2:**

Reviewer #2: This is an interesting paper. I recommend to publish it with a minor revision

**RESPONSE: We thank the reviewer for kind words. We have revised our manuscript based on the comments as described below.**

**Design: Population and sample:**

**REVIEWER COMMENT:** 1. Please explain how were study participants selected? It was purposive, convenience, etc.

**RESPONSE: Thank you for your fruitful comments. We have explained it as follows:**

**A heterogeneous type of purposive sampling strategy was used to recruit the participants. Purposeful sampling is a technique widely used in qualitative research for the selection of information-rich cases, individuals that are experienced with a phenomenon of interest. In addition, it considers the ability to communicate experiences and opinions in an articulate, expressive, and reflective manner [21].**

**A heterogeneous type of purposive sampling technique was used to recruit the participants. The heterogeneous characteristics of participants were maintained by considering age, marital status, educational status, health facilities, and residence, including the most vulnerable young people in the context of Ethiopian priorities and culture. This was done to be able to see the different forms of the lived experience of young epileptic patients from different perspectives.**

**REVIEWER COMMENT:** 2. I suggest to provide information regarding inclusion and exclusion criteria.

**RESPONSE: Thank you for your fruitful comments. We have provided inclusion and exclusion criteria as follows:**

**The participants were eligible for the study if they had been receiving care in the study clinic for at least 6 months duration since diagnosis****.** **Seriously ill patients who are unable to communicate at the time of the data collection period were excluded from the study.**

**Data collection**

**REVIEWER COMMENT:** 3. In which interview data saturation was achieved?

**RESPONSE: Thank you for your fruitful comments.** **We have revised our manuscript by inserting the data about in which interview data saturation was achieved under subheading data collection as:**

**The data saturation was achieved in nineth interview and the additional two interviews were conducted for confirmation of data saturation**.

**REVIEWER COMMENT:** 4. Were transcripts returned to participants for comment or clarification?

**RESPONSE: Thank you for your fruitful comments. We have been invited some of the participants for transcription verification and we have revised it based on your suggestion as follow in credibility part of rigor and trustworthiness section:**

**We also invited 6 participants to review the ideas for transcription verification, which they think the investigator is going to present a true picture from their perspective.**

**Data analysis**

**REVIEWER COMMENT:** 5. Since it is a phenomenological study, please make clear the data analysis method. If thematic analysis, which thematic data analysis steps was adopted?

**RESPONSE: Thank you for your fruitful comments. Our data analysis method was thematic analysis. We have revised it based on your suggestion as:**

**The thematic analysis approach was used to analyse the data. The six thematic data analysis steps were followed to analyze the data such as Step 1: Become familiar with the data, Step 2: Generate initial codes, Step 3: Search for themes, Step 4: Review themes, Step 5: Define themes and Step 6: Write-up [23].**

**Results**

**REVIEWER COMMENT:** 6. In Theme 2 (Subtheme 2) - Troublesome somatic symptoms should be put in theme 1: Psycho-social experience

**RESPONSE: Thank you for your fruitful comments. We have revised it based on your suggestion by moving Troublesome somatic symptoms from Theme 2 (Subtheme 2) to theme 1: Psycho-social experience**
