# Supplementary material for: Lived experience of young people with epilepsy in Bahir Dar city government hospitals, Ethiopia, a qualitative interview study
Source: PLoS One. 2023 Jan 23;18(1):e0278936. doi: 10.1371/journal.pone.0278936 (PMC9870136; doi:10.1371/journal.pone.0278936)
Supplement: Attachment — Submitted filename: Response to Reviewers.docx [file pone.0278936.s005.docx]

**RESPONSE TO EDITOR AND REVIEWERS**

**RESPONSE TO EDITOR**

**RESPONSE: Thank you for coordinating the review process and fruitful comments. We have revised the manuscript and addressed Reviewer’s comments.**

**Journal Requirements:**

**COMMENT:** Please review your reference list to ensure that it is complete and correct. If you have cited papers that have been retracted, please include the rationale for doing so in the manuscript text, or remove these references and replace them with relevant current references. Any changes to the reference list should be mentioned in the rebuttal letter that accompanies your revised manuscript. If you need to cite a retracted article, indicate the article’s retracted status in the References list and also include a citation and full reference for the retraction notice.

**RESPONSE: Thank you for your fruitful comment, we have reviewed our reference list and we can make sure for you that our reference list is complete and correct**

**RESPONSE TO REVIEWER 1:**

**REVIEWER COMMENT:** Reviewer #1:  Thank you for your efforts. Most comments have been addressed. Just some small corrections:
In the abstracts: change, the data were collected through in-depth interview--to semi structured interviews

**RESPONSE: We thank the reviewer for kind words. We have revised it by replacing in-depth interview—by-semi structured interviews**
**REVIEWER COMMENT:** In limitations: add, the limitation of the study was the inability to generalize the findings of this qualitative interview study, but there is opportunities to transfer the findings to similar context and groups of people.

**RESPONSE: We thank the reviewer for kind words. We have revised our limitation section by adding your suggestion as:**

**The limitation of the study was the inability to generalize the findings of this qualitative interview study, but there are opportunities to transfer the findings to similar context and groups of people**

**RESPONSE TO REVIEWER 2:**

**REVIEWER COMMENT:** Reviewer #2:  Thank you so much for your responses to my comments and suggestions. Good luck with your publication.

**RESPONSE: We thank the reviewer for kind words and for your best wish.**
